# Increased Eplet Mismatch Load and Reduced Immunosuppressive Exposure Elevate the Risk of Baseline Lung Allograft Dysfunction

**DOI:** 10.3390/jcm14196864

**Published:** 2025-09-28

**Authors:** Victor M. Mora, Emilio Rodrigo, Elena González-López, Javier Gonzalo Ocejo-Vinyals, David San Segundo, David Iturbe-Fernández, Sheila Izquierdo, Sandra Tello, Marcos López-Hoyos, Maria Mar García-Saiz, Pilar García-Berbel, José M. Cifrián

**Affiliations:** 1 Immunopathology Group, Respiratory Department, Marqués de Valdecilla University Hospital-IDIVAL, University of Cantabria, 39005 Santander, Spain; victormanuel.mora@scsalud.es (V.M.M.); david.iturbe@scsalud.es (D.I.-F.); sheila.izquierdo@scsalud.es (S.I.); sandra.tello@scsalud.es (S.T.); josemanuel.cifrian@scsalud.es (J.M.C.); 2 Immunopathology Group, Nephrology Department, Marqués de Valdecilla University Hospital-IDIVAL, RICORS2040 (ISCIII RD21/0005/0010 and RD24/0004/0019), University of Cantabria, 39005 Santander, Spain; 3 Immunopathology Group, Immunology Department, Marqués de Valdecilla University Hospital-IDIVAL, University of Cantabria, 39005 Santander, Spain; egonzalezlope@saludcastillayleon.es (E.G.-L.); javiergonzalo.ocejo@scsalud.es (J.G.O.-V.); david.sansegundo@scsalud.es (D.S.S.); marcos.lopez@scsalud.es (M.L.-H.); 4 Clinical Pharmacology Department, Marqués de Valdecilla University Hospital-IDIVAL, 39005 Santander, Spain; mmar.garcia@scsalud.es; 5 Pathological Anatomy Department, University Hospital Marqués de Valdecilla, 39008 Santander, Spain; pgberbel@gmail.com

**Keywords:** baseline lung allograft dysfunction, HLA eplet mismatch load, immunosuppressive drug exposure, lung transplantation, mycophenolic acid, tacrolimus

## Abstract

**Background/Objectives:** Some lung transplant (LungTx) recipients do not achieve the expected lung function within the first year, a condition known as baseline lung allograft dysfunction (BLAD). Our objective was to analyze the risk factors associated with BLAD, focusing on the variables associated with a higher risk of developing a more intense alloimmune response. **Methods**: We carried out a prospective study including 88 LungTx recipients. BLAD was defined as failure to reach 80% of the predicted value for forced expiratory volume in one second (FEV1) and/or forced vital capacity (FVC) on two tests conducted at least three weeks apart. Tacrolimus time in therapeutic range (TTR) and mycophenolic acid area under the curve (MPA AUC_0–12h_) were measured at the third month. Donor–recipient compatibility was assessed using HLA eplet mismatch analysis, performed via HLA Matchmaker 3.1. **Results**: BLAD patients showed greater eplet mismatch burden (67, IQR 20 vs. 55, IQR 22, *p* = 0.018) and had been exposed to a lower TTR (26.6%, IQR 14.0% vs. 39.6%, IQR 24.3%, *p* = 0.039) and less frequently to an adequate third-month MPA AUC_0–12_ > 30 mg × h/L (57.1% vs. 89.2%, *p* = 0.020). DR/DQ eplet mismatches (β = −0.348, *p* = 0.002) and third-month MPA AUC_0–12_ (β = 0.285, *p* = 0.009) were independently associated with six-month predicted FEV1%. **Conclusions**: Among other variables, BLAD and initial lung graft function are associated with greater eplet discordance and lower immunosuppressive drug exposure, suggesting a potential role of underlying alloimmune responses in their pathogenesis.

## 1. Introduction

A lung transplant (LungTx) is the preferred treatment option for patients with chronic respiratory diseases who have exhausted all available treatment options and have irreversible respiratory failure that limits their quality of life and life expectancy. The primary goal of lung transplantation is to restore normal lung function, thereby improving both quality of life and survival [1,2]. In most cases, the highest possible lung function is achieved between six and twelve months after transplantation [3]. However, some lung transplant recipients do not achieve the expected lung function within the first year, a condition known as baseline lung allograft dysfunction (BLAD) [4]. The presence of BLAD is linked to a diminished quality of life and an increased risk of chronic lung allograft dysfunction (CLAD) and mortality [4,5,6].

The risk factors associated with partial lung function recovery and BLAD are not yet fully understood. It has been established that certain postoperative recovery variables are associated with an elevated risk of BLAD. These include severe primary graft dysfunction (PGD), mechanical ventilation time, ICU length of stay, initial admission stay length and perioperative blood loss. Furthermore, there is an established correlation between certain donor and recipient variables and BLAD, as well as diminished lung function. These variables include interstitial lung disease as native pulmonary disease, heavy donor smoking, donor age and sex, recipient younger age and recipient obesity [1,2,4,6,7,8,9,10,11].

As BLAD relates to a higher CLAD risk, it is reasonable to hypothesize that both may share some common pathogenic causes. Research has demonstrated a clear association between CLAD and a stronger alloimmune response when considered alongside other variables. Research has indicated that cellular and antibody-mediated rejection episodes, pretransplant and de novo donor-specific antibodies (DSAs), the number of HLA mismatches, and the intensity and adherence to immunosuppressive therapy have been reported as factors associated with CLAD risk [12,13,14,15,16]. It is important to note that some of these variables, such as acute rejection or de novo DSAs, have not been associated with a higher BLAD risk, and their association is controversial. In addition, other variables have not been analyzed in depth [2,17]. Furthermore, various studies have identified discrepancies in the relationship between a well-identified biomarker of alloimmune damage in the lung allograft, such as cell-free DNA, and BLAD [6,18]. The objective of our study was to analyze whether variables associated with a higher risk of developing a more intense alloimmune response, such as greater HLA eplet mismatch number and lower exposure to immunosuppressive drugs, were associated with a higher risk of BLAD.

## 2. Materials and Methods

A prospective study was conducted involving all patients (88, 45 male and 43 female; mean age 60 ± 8 years) who underwent lung transplantation at our center between January 2021 and April 2023 and consented to participate. The study was conducted in accordance with the principles outlined in the Declaration of Helsinki and received approval from the institution’s Regional Ethics Committee (PI20/01710; approved on 22 December 2020). Each participant received a bilateral lung transplant from a deceased donor. Post-transplant immunosuppressive treatment comprised mycophenolate mofetil (1000 mg every 12 h), corticosteroids and tacrolimus, targeting trough levels of 12–15 ng/mL during the first six months. Basiliximab was used as induction therapy, and all patients were given azithromycin as a preventive measure against CLAD.

Data on recipient, donor, transplant-related characteristics and clinical outcomes were collected prospectively from electronic health records (see Table 1). Recipient and donor HLA class-I (A, B, C) and class-II (DR, DQ) typing was performed by a high-resolution sequence-specific primer (Life Technologies, Brown Deer, WI, USA). Donor–recipient compatibility was assessed using HLA eplet mismatch analysis, performed via HLA Matchmaker 3.1 (accessible at http://www.epitopes.net/downloads.html, accessed 1 April 2024). Acute rejection was diagnosed in accordance with the ISHLT Working Formulation, based on findings from transbronchial biopsies [19]. Primary graft dysfunction (PGD) was defined and staged per ISHLT guidelines [20]. Baseline lung allograft dysfunction (BLAD) was defined as failure to reach a minimum of 80% of the predicted value for forced expiratory volume in one second (FEV1) and/or forced vital capacity (FVC) on two tests conducted at least three weeks apart within the first year following transplantation [4].

Tacrolimus trough levels (ng/mL) in whole blood were analyzed using a chemiluminescent microparticle immunoassay on the Architect iSystem (CMIA; Abbott Laboratories, Abbott Park, IL, USA). These levels were recorded up to 90 days post-transplant. The time in therapeutic range (TTR), defined as the proportion of time within 12–15 ng/mL, was calculated using the Rosendaal method [13]. Variability in tacrolimus levels was expressed as the coefficient of variation (CV), using the formula

CV = (μσ) × 100

where σ is the standard deviation and μ is the mean tacrolimus level [21]. The cumulative tacrolimus exposure at three months was estimated by calculating the area under the curve (AUC) of all trough concentrations from the time of transplant to month three [22].

Plasma trough concentrations of mycophenolic acid (MPA) were measured using a homogeneous enzyme immunoassay (Emit 2000; Siemens, Munich, Germany). The full MPA AUC_0–12h_ at three months was estimated using a limited sampling strategy at time points 0, 30 min and 2 h after administration, as described by Pawinski et al. [23]. This measurement was available for 72 patients; 3 declined the procedure and 11 were no longer taking MPA by month three.

Peripheral blood was collected from transplant recipients three months after their transplant using Streck Cell-Free DNA BCT tubes. Plasma was obtained by centrifuging the blood at 1600× *g* for 20 min, followed by 16,000× *g* for a further 10 min. Cell-free DNA was then isolated using a QIAamp Circulating Nucleic Acid Kit (Qiagen, Redwood City, CA, USA). A targeted next-generation sequencing (NGS) panel assessing 202 single-nucleotide polymorphisms (SNPs) (AlloSure^®^, CareDx, Brisbane, CA, USA) was used to determine the relative level of cell-free DNA (cfDNA). Sequencing was performed using Illumina MiSeq equipment, and the results were analyzed using AlloSeq cfDNA software version 1.0 (CareDx). A dd-cfDNA level of ≥1.0% was considered abnormal, in line with the manufacturer’s recommendations.

### Statistical Methods

Continuous variables were summarized using medians and interquartile ranges (IQRs), while categorical variables were reported as proportions. The chi-squared test was used to assess relationships between categorical variables. Spearman’s rank correlation was applied to explore associations between continuous variables. Differences in continuous outcomes between the two groups were tested using the Mann–Whitney U test. Linear regression analyses (both univariate and multivariate) were conducted to identify variables associated with lung function at six months. A *p*-value of less than 0.05 was considered statistically significant. All analyses were performed using SPSS software (version 22.0; SPSS, Inc., Chicago, IL, USA).

## 3. Results

**Figure 1:**
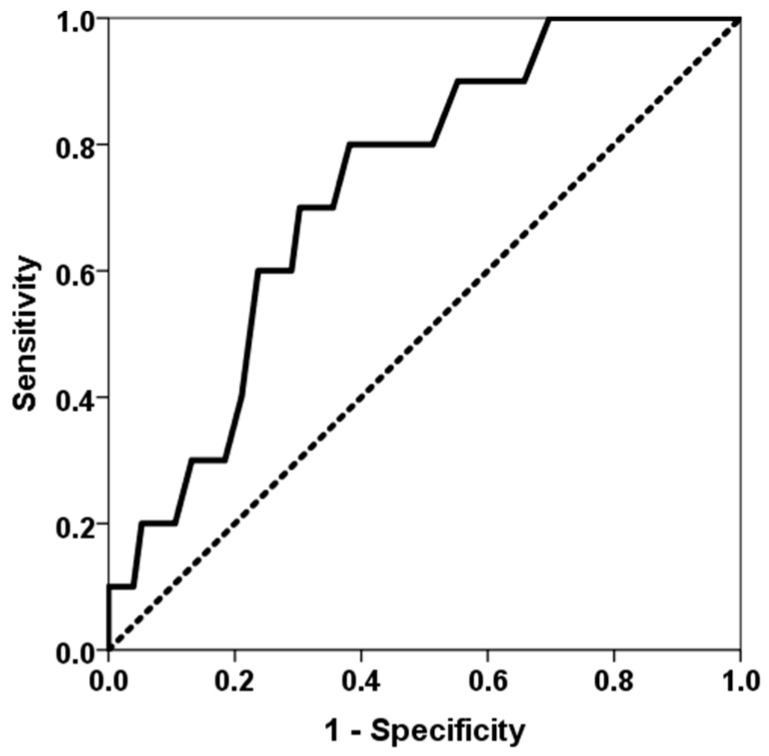
ROC curve of classes-I and –II eplet mismatches for predicting BLAD. The solid line represents the ROC curve (AUC = 0.732). The dotted line represents the reference ROC curve with an AUC of 0.50.

**Figure 2:**
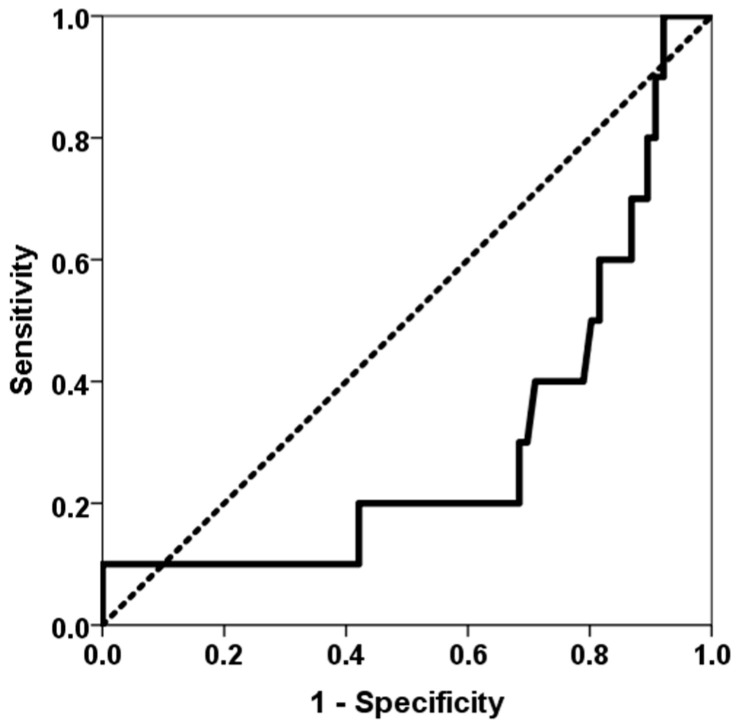
ROC curve of the percentage of time in the therapeutic range for predicting BLAD. The solid line represents the ROC curve (AUC = 0.299). The dotted line represents the reference ROC curve with an AUC of 0.50.

The study included 88 lung transplant recipients who underwent surgery between January 2021 and April 2023. Two patients died during the immediate postoperative period, and ten patients (11.6%) met the criteria for BLAD. The main patient characteristics and their comparison between patients with and without BLAD are shown in Table 1. A higher proportion of BLAD recipients were female, younger and had received more lung grafts from female donors. Patients who developed BLAD experienced longer ventilation times after surgery, as well as longer stays in the ICU and at initial admission. A higher number of class-I and class-II HLA mismatches was associated with a higher BLAD risk (AUC ROC 0.732, 95% CI 0.590–0.873, *p* = 0.018) (Figure 1). Lower time in the therapeutic range of tacrolimus was associated with a higher BLAD risk (AUC ROC 0.299, 95% CI 0.117–0.480, *p* = 0.039) (Figure 2). Furthermore, BLAD patients were less likely to receive an adequate dose of MPA above 30 mg × h/L. As expected, pulmonary function parameters six months after transplantation were significantly lower for patients with BLAD.

**Figure 3:**
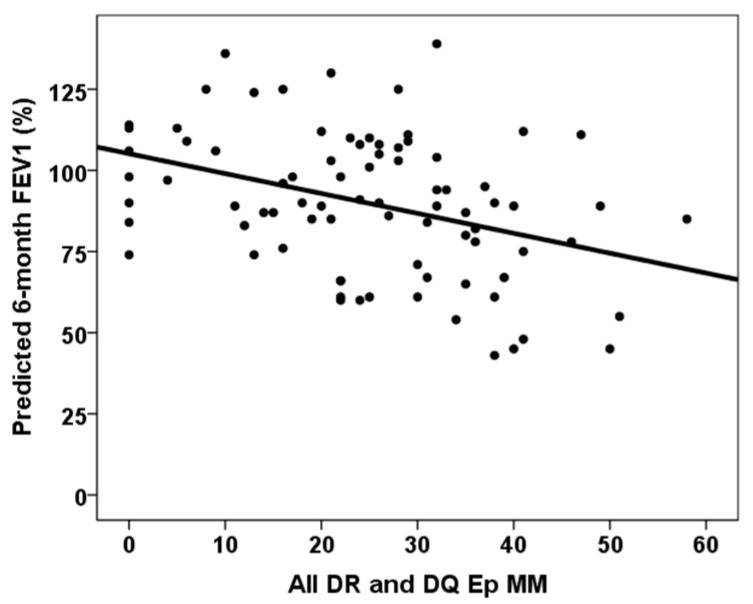
Correlation between six-month predicted FVC% and all DR and DQ eplet mismatches (rho = −0.320, *p* = 0.003).

**Figure 4:**
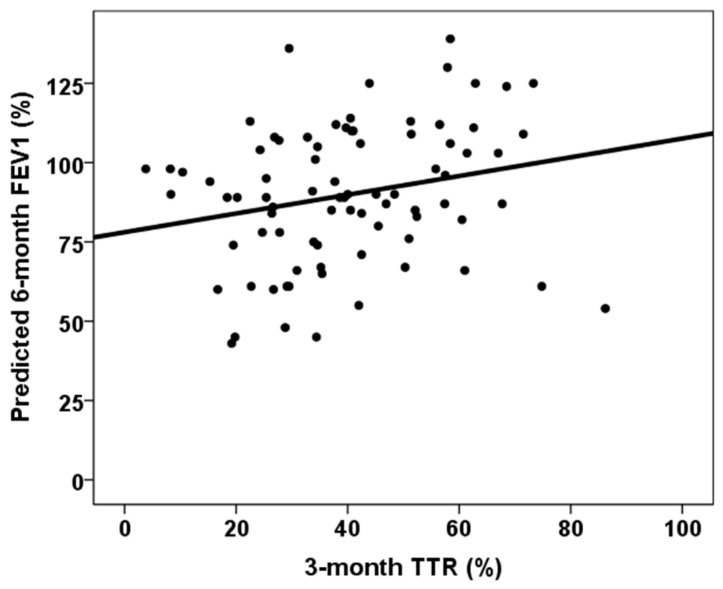
Correlation between six-month predicted FVC% and third-month % time in therapeutic range (rho = 0.383, *p* < 0.001).

**Figure 5:**
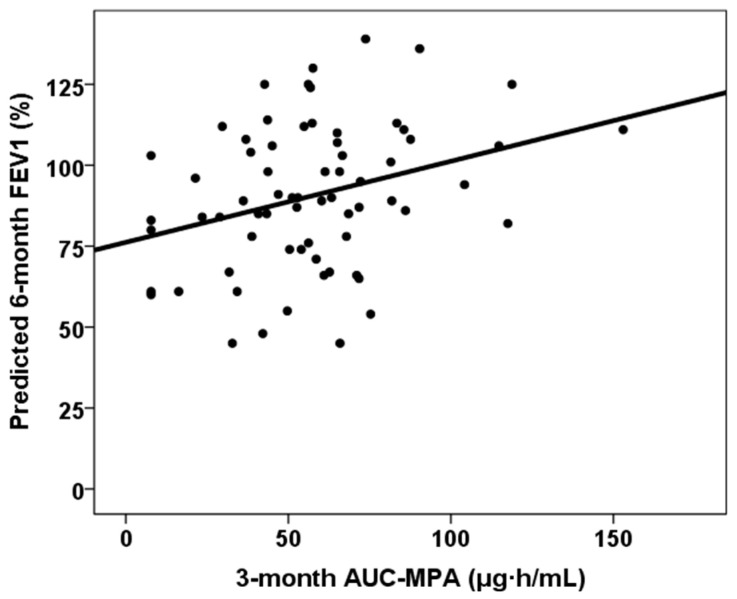
Correlation between six-month predicted FVC% and third-month mycophenolic acid area under the curve (rho = 0.306, *p* = 0.011).

The correlation between continuous variables and six-month predicted forced vital capacity (FVC) and forced expiratory volume in one second (FEV1) is shown in Table 2. All DR and DQ Ep MM, third-month % time in therapeutic range and third-month MPA AUC_0–12_ showed statistically significant correlations with both six-month predicted FVC% and FEV1%. Figure 3, Figure 4 and Figure 5 show the relationship between six-month predicted FVC%, all DR and DQ epitope mismatches, third-month percentage time in therapeutic range and third-month MPA AUC_0–12_, respectively. Female recipients were associated with a lower six-month predicted FVC% (93.0, RIC 30.0 vs. 87.0, RIC 26, *p* = 0.029), but not with a lower six-month predicted FEV1% (90.0, RIC 33 vs. 89.0, RIC 30, *p* = 0.268). In a similar manner, female donors were associated with a lower predicted 6-month FVC% (92.0, RIC 30.0 vs. 87.0, RIC 29, *p* = 0.034), but not with a lower predicted 6-month FEV1% (92.5, RIC 35 vs. 89.0, RIC 31, *p* = 0.296). Patients with cellular rejection and PGD3 did not show worse pulmonary function test results at six months (*p* > 0.05). Lung transplant recipients with COPD as the underlying lung disease showed a higher six-month predicted FVC% (86.0, RIC 31 versus 92.0, RIC 30, *p* = 0.011) and six-month predicted FEV1% (86.0, RIC 29 versus 103.0, RIC 27, *p* = 0.001). Those with ILD as the native lung disease showed a lower predicted FEV1% at six months (97.5, RIC 34 vs. 88.0, RIC 30, *p* = 0.032), but no significant difference in predicted FVC%. Patients in the lowest quartile of TTR showed a lower six-month predicted FVC% (80.5, RIC 27 vs. 92.5, RIC 25, *p* = 0.001), but a similar six-month predicted FEV1% (89.0, RIC 32 vs. 90.0, RIC 34, *p* = 0.103).

Table 3 shows the results of the multivariate linear regression analysis for six-month predicted FVC% and six-month predicted FEV1%. The models included female recipient sex (only for six-month predicted FVC%), interstitial lung disease (ILD) as the native lung disease (only for six-month predicted FEV1%), ventilation length, all donor–recipient DR and DQ epitope mismatches (All DR and DQ EpMM) and time in the therapeutic range. A second model included the MPA AUC_0–12_ at three months for the 72 lung transplant recipients for whom data were available. DR and DQ eplet mismatches and third-month MPA AUC_0–12_ were independently associated with six-month predicted FVC% and FEV1%.

## 4. Discussion

Although the exact causes of BLAD are not fully understood, most studies have found that damage to the lung graft during the initial post-transplant period makes it difficult to achieve maximum lung function. Therefore, consistent with previous studies, we found that patients requiring prolonged mechanical ventilation and ICU and initial hospital stays were at a higher risk of BLAD [2,5,9,11]. However, we did not observe an association between BLAD and a higher proportion of severe PGD or longer second lung ischemia time, in line with the findings of Mohanka et al. and Keller et al. [1,5,6,7,11]. Of the various variables analyzed, we found that younger recipient age increased the risk of developing BLAD, as previously reported [2,6,7]. Due to body size adjustment between donors and recipients during matching, lung grafts from female donors are commonly used for female recipients. We found that both female recipients and donors were associated with a higher BLAD risk. In our series, however, we found no relationship between BLAD and other previously identified variables such as older donor age, interstitial cause of lung disease, and body size of both donor and recipient, probably due to the low number of BLAD cases identified [2,4,5,6,7,11]. Like other authors, we also found no association between BLAD and donor smoking [5,6], although previous studies have observed an association between severe tobacco use (>20 pack-years) and BLAD. Our practice is to accept donors with a lower tobacco burden (<10 pack-years) [4,7,10,11].

Our most significant finding was that variables linked to an increased risk of a more severe alloimmune response, such as greater HLA donor–recipient mismatches and reduced exposure to tacrolimus and MPA immunosuppressive therapy, were associated with an elevated risk of BLAD. Patients with BLAD had a higher number of class-I and class-II mismatches overall, and this epitope discordance load demonstrated the ability to determine whether patients might develop BLAD, with an area under the ROC curve of 73%. However, a previous prospective study by Keller et al. involving 173 lung transplant recipients found that the degree of HLA mismatch at the antigenic level between donor and recipient was not associated with BLAD risk [6]. However, our study showed that analyzing epitope differences increases sensitivity in predicting the appearance of alloimmune responses. Similarly, while several studies have not related BLAD to rejection or DSA development [1,2,6], a more recent study has shown that the appearance of DSAs doubles the risk of BLAD, even in DSA-positive patients without evidence of antibody-mediated rejection (OR 2.14, 95% CI 1.45–3.17, *p* = 0.0001) [17]. Therefore, our findings suggest that BLAD is related not only to initial damage but also to an impending alloimmune response that limits improvement in functional tests. Furthermore, patients with BLAD spent less time within the appropriate tacrolimus therapeutic range (12–15 ng/mL). Notably, patients in the first quartile of time in the therapeutic range (<26.7%) were three times more prevalent in the BLAD group. Similarly, patients with a mycophenolic acid area under the curve of >30 mg × h/L in the third month, which represents a cut-off point associated with adequate exposure to mycophenolate and fewer complications following kidney transplantation, were significantly more prevalent in the non-BLAD group [24]. These findings suggest that more intense immunosuppressive therapy could reduce the incidence of BLAD in cases involving higher HLA mismatch between donor and recipient. However, the small number of BLAD patients limited our ability to perform a multivariate analysis to determine whether these associations were independent of other confounding variables.

To overcome this limitation, we analyzed which variables were associated with predicted pulmonary function tests at month six, which directly relate to the definition of BLAD. As mentioned in Table 2 and Table 3, indicators of greater initial damage, such as a longer duration of mechanical ventilation, ICU and hospital stays, and cold ischemia time, were associated with lower predicted FVC and FEV1 values at month six. These relationships have been reported previously by Yoon et al. and Mohanka et al., and as mentioned above, are explained by residual lung graft damage caused by a complicated early postoperative course [1,8]. Therefore, improving surgical times could contribute to better subsequent lung function and reduce the risk of BLAD. However, the most significant finding of our study was the association between an intense alloimmune response and impaired lung function during the initial year after transplant. In an aforementioned study, Alnababteh et al. reported a relationship between DSAs and CLAD and found that DSA+ lung transplant recipients (most of whom had anti-DQ antibodies) exhibited a significantly lower rate of post-transplant spirometric improvement and declining FVC% and FEV1% slopes after DSA development compared with DSA-negative patients [17]. In line with this finding, we found that greater eplet mismatching, primarily class-II, between donor and recipient increased the risk of poorer lung function at month six independently.

The influence of HLA eplet mismatching on lung transplant outcomes for the development of CLAD, dnDSA and long-term graft survival has been previously reported [16]. However, this influence has not been analyzed for such an early phase of lung grafting. Our findings suggest that measuring eplet mismatch load for all donor–recipient pairs could improve the functional outcomes of lung transplantation by enabling more intensive immunosuppressive strategies to be employed, thereby improving medium- and long-term prognoses.

Importantly, we observed that lung transplant recipients could benefit from greater exposure to MPA than from higher exposure to calcineurin inhibitors. The area under the curve for MPA levels at month three was associated with better lung function test results at month six, independent of the other variables. As previously explained, patients with adequate MPA levels were also less likely to experience BLAD. This relationship was not observed with MMF levels alone, which highlights the importance of measuring the area under the curve for MPA at least once after lung transplantation to optimize treatment and improve lung function during the first year. Although mycophenolic acid and mycophenolate mofetil are usually administered at a fixed dose in routine clinical practice for monitoring solid organ transplant recipients, therapeutic drug monitoring to maintain an MPA AUC_0–12h_ of between 30 and 60 mg × h/L has been shown to prevent both acute rejection and toxicity after kidney transplantation [24]. The relationship between MPA exposure and the course of lung transplantation has not been studied in depth. Interestingly, Yabuki et al. reported that among 59 lung transplant recipients, those with CLAD had been exposed to significantly lower MPA AUC 0–12 values (21 ± 10) than the event-free group (30 ± 7), whereas those with infections had higher values (37 ± 11) [14]. Notably, the authors did not find any relationship between tacrolimus exposure and CLAD development.

Although our data suggest that BLAD patients may be experiencing greater alloimmune damage, we did not find that the percentage of circulating dd-cfDNA, a marker of molecular graft injury, was elevated in BLAD patients, or that higher values were associated with poorer lung function at month six. Similarly to our findings, Keller et al. reported that neither the percentage value of dd-cfDNA nor its reduction over the first 90 days post-transplant was related to a higher risk of BLAD. They argued that BLAD is a highly heterogeneous process influenced by both parenchymal and extraparenchymal lung diseases, as well as the physical state of the patient [6,7]. Interestingly, we found that the percentage of patients with dd-cfDNA values above 1% was almost double in BLAD patients compared with non-BLAD patients. However, this difference was not statistically significant, which could be because of the small number of BLAD patients. Recently, Zajacova et al. reported that BLAD patients had significantly higher absolute dd-cfDNA levels (39 cp/mL vs. 26 cp/mL, *p* = 0.01) than non-BLAD patients. However, the percentage of dd-cfDNA did not differ significantly between the two groups (0.23% vs. 0.15%, *p* = 0.2). The authors demonstrated that absolute dd-cfDNA (OR 3.121, 95% CI 1.444–6.987, *p* = 0.004) but not the percentage of dd-cfDNA had significant predictive value for BLAD [18]. Therefore, subclinical lung allograft injury throughout the first post-transplant year may be associated with BLAD. However, the most effective method of measuring molecular graft injury by analyzing dd-cfDNA (absolute versus percentage) is still under debate.

The main limitation of our study was the small number of patients included, which was partly due to the single-center approach. Nevertheless, we conducted a prospective study focusing on our lung transplant population. For the first time, we analyzed the HLA eplet discordance load between donor and recipient, as well as the continuous exposure to immunosuppressive drugs such as tacrolimus and MPA, in this population. Furthermore, the results were limited by the low incidence of BLAD (12%), which is lower than other studies that reported incidences of greater than 27% [2,4,6,9,11,18]. While we have no definitive explanation for our low BLAD incidence, organ procurement, preservation and surgical procedures are carried out with particular care at our center. In addition, donor–recipient matching is performed with special attention. In two recent studies from our group, we showed that differences in donor–recipient total lung capacity of more than 20% were present in only 10% of cases, and such discrepancies had no impact on survival or the risk of developing CLAD when analyzed separately [25,26]. Moreover, almost 80% of the pairings were sex-matched, which may also contribute to reducing functional mismatches after transplantation. Regarding donor characteristics, nearly two-thirds had never smoked, and the median donor age was 56 years. These data suggest that strict donor selection, with emphasis on age, sex and anthropometric compatibility, could partly explain the relatively low incidence of BLAD observed in our cohort. Additionally, all our lung transplant recipients received azithromycin prophylaxis, which reduces the risk of BLAD by almost half (OR 0.53, 95% CI 0.33–0.85, *p* = 0.046) [27,28]. Our study links the presence of BLAD with lower exposure to immunosuppressive drugs and a higher burden of HLA mismatch between donor and recipient, suggesting that alloimmunity mechanisms may contribute to its development. Consequently, another relevant limitation is the absence of a systematic histological study to determine whether patients with BLAD experience more underlying rejection. Finally, because of our findings, it would have been very interesting to record tacrolimus blood levels beyond the third month to know its influence on the appearance of BLAD throughout the first-year post-transplant.

## 5. Conclusions

In conclusion, our study is the first to analyze the influence of donor–recipient mismatch and exposure to immunosuppressive drugs on the risk of BLAD and poor early lung graft function. Our findings suggest that BLAD is related not only to early graft damage but also to an underlying alloimmune response. We found that among other variables, greater eplet discordance, especially class-II, was associated with a higher risk of BLAD, as well as worse predicted FVC% and FEV1% at month six. Furthermore, we observed, for the first time, that higher MPA exposure was associated with a lower risk of BLAD and better subsequent lung function. If confirmed in subsequent studies, these findings highlight the importance of determining eplet discordance in lung transplant recipients to optimize immunosuppressive therapy for those with greater discordance. One potential strategy could be to specifically measure the area under the MPA curve to identify those with low MPA exposure who are at a higher risk of BLAD and poorer lung function and could benefit from dose adjustment by MPA AUC_0–12h_.

## Figures and Tables

**Table 1:** Recipient, donor and transplant characteristics.

	No BLAD = 76	BLAD = 10	*p*-Value
Recipient age (years)	63 (IQR 8)	56 (IQR 21)	0.021
Female recipient	44.7%	80.0%	0.036
BMI (kg/m^2^)	24.5 (IQR 4.3)	25.4 (IQR 6.0)	0.903
Recipient height (cm)	165 (IQR 14)	163 (IQR 8)	0.594
Recipient weight (kg)	67 (IQR 16)	64 (IQR 17)	0.666
Native lung disease: ILD	47.4%	50.0%	0.876
Native lung disease: COPD	43.4%	30.0%	0.419
LAS	33.5 (IQR 2.6)	33.9 (IQR 4.5)	0.657
Ventilation length (days)	1.0 (IQR 0.1)	2.0 (IQR 11.0)	0.028
ICU stay length (days)	3.0 (IQR 2.0)	10.0 (IQR 28.0)	0.007
Hospitalization stay length (days)	21.0 (IQR 9.0)	32.0 (IQR 45.0)	0.027
PGD grade 3 at 0–72 h	9.2%	10.0%	0.936
Ischemic time (min)	373 (IQR 95)	396 (IQR 104)	0.220
Donor age	55 (IQR 19)	59 (IQR 6)	0.505
Female donor	43.4%	80.0%	0.029
Donor smoking history	68.5%	50.0%	0.246
Donor height (cm)	168 (IQR 15)	166 (IQR 10)	0.746
Donor weight (kg)	73 (IQR 20)	70 (IQR 22)	0.622
All class-I Ep MM	29.0 (IQR 15.0)	35.0 (IQR 16.0)	0.054
All DRB1 Ep MM	14.0 (IQR 14.0)	17.5 (IQR 20.0)	0.151
All DQ Ep MM	10.0 (IQR 8.0)	13.0 (IQR 11.0)	0.113
All DR and DQ EpMM	25.2 (IQR 19.5)	28.0 (IQR 19.0)	0.133
All class-I and II Ep MM	55 (IQR 22)	67 (IQR 20)	0.018
Cellular rejection	35.5%	20.0%	0.329
Third-month dd-cfDNA *	0.39 (IQR 0.74)	0.51 (IQR 4.16)	0.499
Third-month dd-cfDNA > 1% *	22.2%	40.0%	0.371
Six-month FVC (ml)	3190 (IQR 1525)	2110 (IQR 1005)	<0.001
Six-month predicted FVC%	90.5 (IQR 22.0)	59.0 (IQR 11.0)	<0.001
Six-month FEV1 (ml)	2490 (IQR 1253)	1655 (IQR 735)	<0.001
Six-month predicted FEV1%	92.5 (IQR 26.0)	60.0 (IQR 11.0)	<0.001
Third-month % time in therapeutic range	39.6 (IQR 24.3)	26.6 (IQR 14.0)	0.039
Third-month % time in therapeutic range below first quartile	21.1%	60.0%	0.008
Total tacrolimus exposure at third month	1096 (IQR 140)	1075 (IQR 234)	0.649
Third-month tacrolimus CV	42.0 (IQR 8.7)	43.0 (IQR 7.4)	0.957
Third-month MPA AUC_0–12_ **	56.8 (IQR 29.9)	32.8 (IQR 44.7)	0.069
Third-month MPA AUC_0–12_ > 30 mg × h/L **	89.2%	57.1%	0.020

BLAD, baseline lung allograft dysfunction; BMI, body mass index; ILD, interstitial lung disease; COPD, chronic obstructive pulmonary disease; PGD, primary graft dysfunction; LAS, lung allocation score; ICU, intensive care unit; FVC, forced vital capacity; FEV1, forced expiratory volume in one second; MM, mismatch; Ep, eplet; CV, coefficient of variability; MPA AUC_0–12,_ mycophenolic acid area under the curve. * Available for 59 patients; ** available for 72 patients.

**Table 2:** Spearman’s correlation between continuous variables and pulmonary function parameters at six months after transplantation.

	Six-Month Predicted FVC%	Six-Month Predicted FEV1%
Recipient age (years)	rho = 0.125, *p* = 0.262	rho = 0.093, *p* = 0.406
BMI (kg/m^2^)	rho = −0.081, *p* = 0.469	rho = −0.171, *p* = 0.124
Recipient height (cm)	rho = 0.131, *p* = 0.240	rho = 0.036, *p* = 0.751
Recipient weight (kg)	rho = 0.019, *p* = 0.869	rho = −0.112, *p* = 0.317
LAS	rho = −0.115, *p* = 0.305	rho = −0.258, *p* = 0.020
Ventilation length (days)	rho = −0.360, *p* = 0.001	rho = −0.324, *p* = 0.004
ICU stay length (days)	rho = −0.204, *p* = 0.066	rho = −0.263, *p* = 0.017
Hospitalization stay length (days)	rho = −0.285, *p* = 0.009	rho = −0.215, *p* = 0.053
Ischemic time (min)	rho = −0.294, *p* = 0.007	rho = −0.189, *p* = 0.088
Donor age	rho = 0.094, *p* = 0.402	rho = −0.120, *p* = 0.283
Donor height (cm)	rho = 0.177, *p* = 0.112	rho = 0.050, *p* = 0.654
Donor weight (kg)	rho = 0.051, *p* = 0.652	rho = −0.078, *p* = 0.484
All class-I Ep MM	rho = −0.062, *p* = 0.577	rho = 0.019, *p* = 0.868
All DR Ep MM	rho = −0.281, *p* = 0.010	rho = −0.303, *p* = 0.006
All DQ Ep MM	rho = −0.265, *p* = 0.016	rho = −0.338, *p* = 0.002
All DR and DQ Ep MM	rho = −0.320, *p* = 0.003	rho = −0.369, *p* = 0.001
All classes-I and -II Ep MM	rho = −0.308, *p* = 0.005	rho = −0.315, *p* = 0.004
Third-month dd-cfDNA	rho = −0.108, *p* = 0.429	rho = −0.069, *p* = 0.615
Third-month % time in therapeutic range	rho = 0.383, *p* < 0.001	rho = 0.251, *p* = 0.023
Total tacrolimus exposure at third month	rho = 0.062, *p* = 0.585	rho = 0.055, *p* = 0.630
Third-month tacrolimus CV	rho = −0.083, *p* = 0.460	rho = −0.013, *p* = 0.905
Third-month MPA AUC_0–12_	rho = 0.306, *p* = 0.011	rho = 0.292, *p* = 0.016

**Table 3:** Multivariate linear regression analysis for pulmonary function parameters at six months after transplantation.

	Six-Month Predicted FVC%	Six-Month Predicted FVC%	Six-Month Predicted FEV1%	Six-Month Predicted FEV1%
Female recipient	β = −0.327, *p* = 0.019	β = −0.288, *p* = 0.065	-	-
ILD	-	-	β = −0.249, *p* = 0.020	β = −0.282, *p* = 0.011
Recipient height (cm)	β = −0.171, *p* = 0.225	β = −0.085, *p* = 0.589	β = −0.005, *p* = 0.960	β = 0.031, *p* = 0.779
Ventilation length (days)	β = −0.327, *p* = 0.002	β = −0.276, *p* = 0.012	β = −0.242, *p* = 0.023	β = −0.164, *p* = 0.134
All DR and DQ EpMM	β = −0.338, *p* = 0.001	β = −0.340, *p* = 0.002	β = −0.311, *p* = 0.004	β = −0.348, *p* = 0.002
Third-month % TTR	β = 0.172, *p* = 0.090	β = 0.093, *p* = 0.395	β = 0.067, *p* = 0.530	β =- 0.003, *p* = 0.981
Third-month MPA AUC_0–12_	-	β = 0.254, *p* = 0.018	-	β = 0.285, *p* = 0.009

## Data Availability

The data presented in this study are available on request from the corresponding author.

## Abbreviations

The following abbreviations are used in this manuscript:

BLAD	Baseline lung allograft dysfunction
BMI	Body mass index
CLAD	Chronic lung allograft dysfunction
COPD	Chronic obstructive pulmonary disease
CV	Coefficient of variability
DSA	Donor-specific antibody
Ep	Eplet
FEV1	Forced expiratory volume in one second
FVC	Forced vital capacity
ICU	Intensive care unit
ILD	Interstitial lung disease
IQR	Interquartile range
LAS	Lung allocation score
LungTx	Lung transplant
MM	Mismatch
MPA	Mycophenolic acid
MPA AUC_0–12_	Mycophenolic acid area under the curve
PGD	Primary graft dysfunction
TTR	Time in therapeutic range
